# Supplementary material for: Are carbon emissions trading and green financial instruments synergistic? -Comprehensive quantitative research based on content analysis
Source: PLoS One. 2024 Mar 7;19(3):e0298601. doi: 10.1371/journal.pone.0298601 (PMC10919601; doi:10.1371/journal.pone.0298601)
Supplement: S1 Data — (ZIP) [file pone.0298601.s001.zip › Data/地级市Z_F工作报告.zip!/╕ú╜¿/─╧╞╜╩╨/└┤╫╘╣1⁄2╓┌║┼í╛┬φ┐╦╩2╛▌═°í┐_2013.docx]

　各位代表：

　　现在，我代表南平市人民政府向大会作政府工作报告，请予审议，并请市政协各位委员和其他列席的同志提出意见。

　　一、2012年工作回顾

　　2012年，在省委、省政府和市委的正确领导下，我市各级政府认真学习贯彻党的十七大、十八大和省委九届五次、六次全会，市委四届三次、四次全会精神，深入贯彻落实科学发展观，积极应对宏观环境趋紧、经济下行压力增大的复杂形势，有效克服雨水偏多影响项目建设等不利因素，全力打好“五大战役”，加快推进绿色发展，基本完成市四届人大一次会议确定的年度目标任务，实现了本届政府良好开局。

　　（一）积极拓思路求转变，绿色发展态势良好。站位海西发展全局，进一步深化拓展海西绿色腹地内涵，提出绿色发展思路，启动实施绿色发展示范项目，大力发展绿色经济、打造绿色城乡、倡导绿色消费、保护绿色生态，积极创建全国绿色发展示范区，经济保持持续较快增长的良好态势。初步统计，2012年全市生产总值997亿元，增长11%；财政总收入92亿元，增长18.9%，其中地方级财政收入59.2亿元，增长21.6%；规模以上工业总产值1120亿元，现价增长20%；农林牧渔业总产值386亿元，增长5.6%；全社会固定资产投资899亿元，增长30%；验资口径实际利用外资8700万美元，增长12%；实际利用内资94亿元，增长30%；外贸出口16.9亿美元，增长41.6%；社会消费品零售总额346亿元，增长16.7%；城镇居民人均可支配收入22235元，增长12.7%；农民人均纯收入8893元，增长13.1%；居民消费价格总水平上涨2.4%。

　　（二）全力抓重点攻难点，“五大战役”成效明显。突出重点工作重大项目，加强要素保障和运行调度，全力破解融资难、征迁难、人才不足等问题，保障了重点建设顺利进行。全市20项重点工作、38个重大项目扎实推进，“五大战役”实施项目1230个，超额完成年度目标。融资渠道有效拓展，争取地方政府债券8.27亿元，武夷新区与金融机构签署530亿元的战略合作框架协议，整合市本级国有资产，新组建南平建设集团等4家国有集团公司。争取上级各类补助资金84亿元，增长25.5%。现代交通体系不断完善，龙浦、宁武、松建三条高速公路全面建成通车，8个县（市、区）通高速公路，通车里程757公里，跃居全省首位；京福高铁南平段加快建设，向莆铁路南平连接线外洋段竣工通车。城乡面貌明显改观，南平中心城市延平新城启动建设，闽江大桥建成通车，交通秩序整治取得成效；县（市、区）“六个一”项目扎实推进，一批旧城改造、新城建设项目加快建设；4个省级和10个市级小城镇改革试点工作顺利实施，180个中心村加快建设，20个绿色试点村启动建设。

　　（三）突出抓龙头铸链条，产业升级步伐加快。深化产业梳理，启动实施食品、旅游产业千亿产值行动计划，食品加工、旅游、机电制造、竹木加工、纺织箱包等传统产业进一步整合提升，生物医药、光电、文化创意等新兴产业加快培育。落实国家和省上扶持企业发展政策，整合工业发展基金，出台推进企业技术改造措施，促进中小微企业发展。实施155项重点工业在建项目，推动现有企业增资扩产，顺昌欧浦登光学扩建项目竣工投产，圣农集团在光泽扩建项目部分投产，在浦城、松溪、政和新建项目开工建设，福矛窖酒、青松化工、和泉生物等企业生产线扩建项目进展顺利，产值超亿元企业257家、净增57家。

　　（上接第1版）市本级三大产业组团完成投资57亿元，引入企业76家，新增工业产值37亿元。各县（市、区）工业园区加快建设。实施品牌战略成效明显，新创中国驰名商标5件、福建省著名商标41件、地理标志证明商标4件、境外商标注册34件。武夷山欢乐茶城、邵武瀑布林温泉等一批旅游项目相继建成，武夷山杜坝旅游服务园区、武夷千古情城、邵武和平古镇等12个旅游综合体和20个高星级饭店项目开工建设，武夷山国家旅游标准化和智慧旅游试点城市工作有序推进，旅游接待总人数1868万人次，旅游总收入223亿元，分别增长22.9%、22.8%。文化产业加快发展，武夷山创意园区等15个重点文化产业项目开工建设。

　　（四）坚持大招商招大商，“三维”项目对接有力。紧紧把握武夷新区建设和交通条件改善的机遇，加大对外招商推介力度，组织赴北京、上海、深圳、福州、厦门、香港、台湾、东南亚等地开展招商推介，积极参加“6·18”、“9·8”等重大展会，成功举办“5·13”投洽会、茶博会等重大经贸活动，大力推进与央企、民企、外企项目对接，加强与省属国有企业全方位合作，引进项目410个、总投资1900多亿元，其中对接“三维”合同项目160个、总投资850亿元。坚持抓招商与抓项目落地并重，建立招商项目集中审批制度，实行招商工作和项目审批代办“一条龙”服务，全年签约项目开工率达68%。

　　（五）立足高起点高标准，新区建设全面展开。坚持规划先行，武夷新区城市总体规划获省政府批准实施，完成武夷东站片区等5个控制性详规和绿道网等7个专项规划编制。启动实施 “十大工程、百亿投资”战役，南林核心区场平工程全面展开，滨江路一期景观绿化、林后大桥等10个市政基础设施项目竣工。新区入驻产业项目85个，总投资270亿元，其中超亿元投资项目50个，兴华啤酒、派森家居、荣田钢构等25个项目建成投产，海源新材料等5个投资超10亿元的项目开工建设，长河动力锂电池产业基地、生物医药产业园等一批项目已签约。

　　（六）持续强基础创特色，“三农”工作扎实推进。落实强农惠农富农政策，粮食生产保持稳定，主要农产品增产增收。农业产业化加快推进，省级以上农业产业化重点龙头企业达34家，新增农民专业合作社115家，延平王台现代农业示范区、浦城丹桂示范园等加快建设。农业“五新”示范推广290项，农业机械化水平达40%。全市涉农乡镇（街道）全部建成土地流转服务平台，土地流转52万亩。实施土地整理、高标准农田建设和中低产田改造9万亩。基本完成农村集体土地确权登记发证工作。新开工重点水利项目23个，水库除险加固73座，解决30万农村人口饮水安全问题，水利普查工作顺利完成。治理水土流失30万亩。造林绿化47万亩。硬化农村公路320公里，实施安保2100公里，改造危桥138座。5个省级扶贫开发重点县对接项目270个，落实资金23亿元。完成农村造福工程危房改造9400户、3.6万人，建成18个省级集中安置区。“南平机制”深化拓展，基本完成村级组织换届选举，建设省级“三农”服务中心30个。

　　（七）着力惠民生促和谐，社会建设取得进步。市委、市政府确定的50件为民办实事项目全面完成。支持创业促进就业，新增城镇就业2.3万人，农村劳动力转移就业5.2万人，城镇登记失业率3.5%。稳步推进社会保障体系建设，城乡居民基本养老保险实现全覆盖，基本医疗保险制度改革持续深化。保障性安居工程开工建设9600套、基本建成8500套，超额完成年度任务。新建松溪县社会福利中心、12个社区综合服务站、115个社区居家养老服务站和12所农村敬老院。启动智慧城市建设，成功举办第五届科技成果交易会，科技成果转化能力提升，知识产权工作不断强化。人才强市战略扎实推进，人才发展环境不断优化。群众性精神文明创建活动持续深化，学习宣传詹红荔、朱邦月等先进典型兴起新高潮。落实教育惠民政策，竣工274个校舍安全工程项目，新建23所公办幼儿园，创建103所义务教育标准化学校，实现义务教育初步均衡。加快构建公共文化服务体系，县级公共图书馆和城区影院、群众性文化激情广场示范点、农家书屋等文化惠民工程建设取得成效。深化医药卫生体制改革，公立医院试点改革稳步实施，完成460个建制村卫生所标准化建设，基本药物制度覆盖到全市基层医疗机构。“生育文明·幸福家庭”促进计划全面启动，人口自然增长率控制在6.5‰以内。成功举办第二届市运会，群众体育和竞技体育运动蓬勃开展。生态市建设加快推进，重点流域水环境综合整治、农村家园清洁行动和环境连片整治、重要水源地保护力度加大，建阳垃圾焚烧发电厂投入运营，新创建省级生态县（市）4个、生态乡镇30个、生态村113个，我市被列入全国第二批低碳试点城市。妇女儿童、老龄和残疾人工作得到加强，社会福利和慈善事业稳步发展。深化“平安南平”建设，扎实开展领导干部接访下访活动，信访积案有效化解，综合警务改革创新工作取得成效，社会保持安定稳定。开展道路交通安全集中整治大会战和消防安全、食品药品安全等专项行动，安全生产形势总体稳定好转。民族宗教、外事侨务、气象防震、档案方志、老区库区等工作继续加强。军政军民团结巩固发展，第三次蝉联“全国双拥模范城”称号。

　　（八）注重转作风提效能，政府自身建设得到加强。坚持依法行政，自觉接受人大及其常委会的监督和政协的民主监督，办理市人大代表议案2件、建议207件，市政协委员提案278件，办复率100%。广泛听取民主党派、工商联、无党派人士和社会各界人士意见。深化行政审批制度改革，全面推行电子政务，建成市本级网上行政执法平台，基本建成县（市、区）行政服务中心和公共资源交易平台，行政权力运行不断规范。深入开展“大干180天、全力推进五大战役”活动，推行一线工作法、“五张表格”管理法、“五个一”项目推进法，大力整治庸懒散奢行为，机关效能建设持续深化，服务质量明显提升。加强重点领域、重点项目、重点资金审计监督，专项治理公务用车，廉政建设和反腐败工作扎实推进。

　　这些成绩的取得来之不易，是省委、省政府正确领导和省直部门鼎力支持的结果，是市委科学决策，带领全市人民凝心聚力、奋勇拼搏的结果，是全市人大代表、政协委员、各民主党派、工商联、各人民团体、无党派人士、离退休老同志和社会各界人士有效监督、大力支持的结果，是对口帮扶地区热心支持的结果，是中央、省属驻南平机构和闽北驻军、武警官兵、公安干警无私奉献的结果，是港澳台同胞、海外侨胞、国际友人关心支持的结果。在此，我代表市人民政府表示衷心的感谢！

　　在看到成绩的同时，我们也清醒地认识到存在的问题和不足。主要表现在：经济总量较小，产业结构不够合理，商贸等第三产业发展相对滞后，部分中小微企业经营困难；引进的大项目、好项目不多，发展后劲不足；城乡规划建设管理水平有待提高，基础设施建设任务依然较重，抵御自然灾害能力较弱；公共服务设施欠账较多，优质教育、医疗资源总量不足、分布不均；节能减排、生态环境保护、安全生产压力增大；一些干部服务意识不强，办事效率不高，消极腐败现象和不正之风在一些领域仍然存在。我们必须直面这些问题，切实采取有效措施加以解决。

　　二、2013年发展目标任务

　　2013年，是我市全面贯彻落实党的十八大精神、加快推进绿色发展、实施“十二五”规划的关键一年。政府工作的总体要求是：深入学习和全面贯彻落实党的十八大精神，坚持以邓小平理论、“三个代表”重要思想、科学发展观为指导，积极融入海西发展大局，按照市第四次党代会的部署，紧紧围绕主题主线，以提高经济增长质量和效益为中心，稳中求进、好中求快，大力实施绿色发展战略，推进四化同步，加强三维对接，主动迎接高铁时代，继续打好“五大战役”，在加快建设美丽南平上取得更大的成效。

　　今年经济社会发展的主要预期目标是：全市生产总值增长11.5%；财政总收入和地方级财政收入分别增长13%；全社会固定资产投资增长20%以上；外贸出口增长6%，验资口径实际利用外资增长10%，实际利用内资增长15%；旅游接待人数和收入分别增长20%和26%；城镇登记失业率控制在4.2%以内；社会消费品零售总额增长15%；居民消费价格总水平涨幅控制在3.5%左右；人口自然增长率控制在7.1‰以内；城镇居民人均可支配收入增长11.5%，农民人均纯收入增长12%；完成单位生产总值能耗、化学需氧量、二氧化硫、氨氮、氮氧化物等年度节能减排任务。在实际执行中，力求更好更快一些。

　　确定上述发展目标，是实现“十二五”规划、全面建成小康社会的客观需要，也是基于对发展形势的科学判断。随着国家和省委、省政府支持南平绿色发展、支持武夷新区开发建设的力度不断加大，我市现代立体交通体系的不断完善，特别是高铁时代即将到来，加快发展迎来了重大历史性机遇。我们要正确把握形势，抢抓发展机遇，力求更大作为，实现更快发展。具体工作中，我们必须把握好以下原则：一是坚持绿色发展。把生态文明建设放在突出位置，坚持规划引领，突出示范带动，着力打造全国绿色发展示范区。二是坚持投资拉动。善于借势借力，策划大项目，持续招大商，争取大资金，以大投入拉动大发展。三是坚持产业支撑。大力发展实体经济，加快培育千亿产业、五百亿产业和百亿企业集团，做大经济总量。四是坚持城乡统筹。加快建设绿色城市和美丽乡村，提高城镇化质量，推进城乡一体化。五是坚持改革创新。进一步解放思想、更新观念，用改革的办法和创新的举措破解发展难题，激发创新发展新动力。六是坚持民生优先。着力在加快发展中改善民生，在创新管理中加强社会建设，更好地让全市人民共享改革发展成果。

　　（一）主动迎接高铁时代，努力扩大投资增后劲

　　加大项目策划力度。围绕绿色发展目标，策划实施一批有特色、有内容、可推广的绿色发展示范项目，切实发挥中央、省上扶持南平发展的政策效应。认真组织实施迎接高铁时代到来行动方案，提前谋划并大力推进与高铁配套衔接的交通、旅游、城建、物流等项目建设，确保与高铁同步投入使用。做深做细项目前期，积极争取更多的项目和资金支持，努力形成储备一批、开工一批、投产一批的项目滚动发展格局。

　　深入开展招商选资。加强综合性招商，积极参加“6·18”、“9·8”和民企洽谈会等重大展会，办好“5·13”投洽会和茶博会，提高市场化水平和客商参会率。突出专业性招商，依托专业园区，通过行业协会以商招商，大力引进行业龙头企业、上市公司以及有意向、有潜力的投资者，引导外地闽北客商回归。深化“三维”对接，加强与央企、省属国有企业、全国百强民营企业和世界500强企业的合作，力求引进更多的大项目、好项目。落实招商优惠政策，强化重大项目跟踪服务，促成项目尽快落地投产。

　　继续打好“五大战役”。拓展战役的内涵和内容，把现代服务业、“六个一”项目、县（市、区）新增长区域、生态建设、“数字南平”等领域项目纳入战役组织实施。提升推动战役的方法和措施，坚持重点工作重点突破，强化组织实施调度，多方拓展融资渠道，全面推进和谐征迁，推广标准化管理，推进质量、效益、安全、进度、廉政“五同步”。扩大战役投资规模，努力做到重点项目建设战役投资与全社会固定资产投资同步增长，新增长区域、城市建设、小城镇改革发展、民生工程等战役投资高于去年。

　　强化基础设施建设。加快建设京福高铁南平段、南三龙铁路南平段，建成向莆铁路南平连接线，推进浦建龙梅铁路、衢宁铁路南平段前期工作。加快建设京台高速公路南平段、邵光高速公路，开工建设延顺、顺邵、南平联络线高速公路。实施新一轮普通公路路网规划改造建设，提升公路通行等级。加快武夷山机场改扩建项目建设，推进武夷山新机场项目前期工作。强化电力能源保障，持续推进农网升级改造工程，续建220千伏变电站3座，新扩建110千伏变电站10座，争取开工建设邵武火电厂扩建项目，加快推进天然气管网工程建设。

　　（二）着力推进转型升级，提升优势产业竞争力

　　加快推进新型工业化。持续突出工业突破工业，通过抓龙头、铸链条、建集群，推动主导产业高端化、传统产业新型化、新兴产业规模化。组织实施食品产业千亿产值行动计划，大力支持现有龙头企业加快发展，着力引进一批大企业，延伸产业链条，力争产值增长30%以上。落实用电奖励、技改贴息、企业减负等政策，发挥企业帮扶基金作用，支持有市场、有效益的企业通过上市、发债、引进战略投资者等方式实现增资扩产，实施投资5000万元以上重点工业技改项目150项以上。加快建设县（市、区）专业园区，明确园区定位和产业发展方向，重点发展1—2个特色主导产业，严格项目准入，同步建设污水处理设施和公共服务配套设施。创新市本级三大组团运作机制，形成由园区管委会、当地政府、市土地总公司、市属国有集团公司分工负责、统一运作的格局，力争新增产值50亿元以上。

　　发展大武夷旅游经济。实施旅游产业千亿产值行动计划，促进文化、农业、工业、城乡建设与旅游业融合发展，延伸旅游产业链条。加快推进紫阳古城旅游综合体等28个旅游重点项目建设，支持武夷山创建国家首批生态旅游示范区，完善各县（市、区）旅游配套设施，提升旅游接待能力。整合县（市、区）旅游资源，打造精品路线，创新促销模式，培育“大武夷”旅游套餐。深化旅游区域协作，加大航班培育力度，增开旅游专列和经停列车，拓展客源市场。推进旅游标准化和信息化建设，建设武夷山智慧旅游城市，加强旅游市场管理，不断优化旅游环境。

　　培育现代服务业。大力引进国内外大型知名品牌商贸企业，加快推进海西蔬果茶交易中心等29个服务业重点项目建设，积极发展现代物流、电子商务、信息服务、服务外包等生产性服务业，引进培育技术、信息、人才、法律、财务审计等中介服务业。改造提升城乡消费设施和服务体系，合理布局建设一批大型超市、农贸市场、专业市场和便民生活服务网点。加快发展金融服务业，引导更多的股份制商业银行进驻我市设立分支机构，发展村镇银行，培育金融租赁等非银行金融机构，探索成立产业投资基金。加强金融监管，防范金融风险。大力培育中心商务区和总部经济，扶持会展业发展。

　　（三）加快发展现代农业，建设社会主义新农村

　　培育农业特色产业。坚持用工业化理念发展农业、用现代科学技术提升农业、用现代经营方式拓展农业，加快“十个一批”现代农业示范项目建设，促进农业增效、农民增收。全面落实稳粮措施，建设“菜篮子”基地，确保粮食安全和重要农产品有效供给。立足“一县一特色”，因地制宜推广种植丹桂、桔柚、百合花等地理标志产品和特色农产品，加快发展茶叶、烟叶、笋竹、无患子、油茶等产业，扶持龙头企业建立生产基地，促进农产品就地深加工，新增规模以上农产品加工龙头企业20家。扶持花卉苗木和林下经济发展，大力培育农家乐、森林人家、水上渔村等休闲观光农业，打造一批集农产品种植加工、展示营销、休闲旅游为一体的农庄、茶庄、果庄。加强无公害、绿色和有机农产品认证，推进农业标准化生产，严格农产品质量安全监管。

　　改善农村生产生活条件。主动对接全省大水网规划，加快重大水利工程建设，开工建设闽江南平段防洪工程。实施水功能区划，开展重点地区中小河流治理，抓好30座小型病险水库除险加固，解决30万农村人口饮水安全问题。实施农业综合开发，建设烟叶水源工程，加快推进土地整理、中低产田改造和高标准农田建设。实施农村公路安保1600公里，完成危桥改造100座。加强农业科技推广应用，探索成立武夷山茶产业技术创新联盟，推进国家级闽北星火技术产业带建设，加快推进农业机械化，建立农业“五新”示范片（场）100个。完善农村气象灾害防御体系，扩大农业政策性保险覆盖面，提高防灾减灾能力。

　　增强扶贫开发实效。主动对接中央和省上扶贫开发相关政策，抓紧做好项目前期工作，加快建设山海协作共建产业示范园，抓好项目落实，推动省级扶贫开发重点县经济发展。支持原中央苏区、革命老区、少数民族地区、偏远山区和库区发展，推进整村帮扶，开展产业扶贫，增强贫困地区自我“造血”功能。

　　创新农业生产经营体制。健全农村土地流转制度，完善土地流转服务平台，发展多种形式的适度规模经营。着力培育新型经营主体，培养新型农民，规范提升农民合作社。持续深化“南平机制”，充分发挥科技特派员和“六大员”作用，提高农村公共服务能力。推广农作物病虫害统防统治模式，支持农业企业、合作社、专业协会等开展多种形式的农业社会化服务。

　　（四）精心建设优美城乡，推进城乡发展一体化

　　加强城乡规划管理。围绕建设山水相融、城景相依的组团式山水园林城市和美丽乡村，完善城乡规划体系，抓好城乡风貌、景观和建筑设计，把闽北文化元素融入到建筑中，彰显特色和品位，争取把每一处新建的公共设施都建成旅游景观、把每一座城市都建成旅游景区。加强城市综合执法，整治违法建设，完善社区服务功能，提高城市管理水平。优先发展城市公交，增加公交车辆和公交站点、线路，完善公共停车场、立交桥等配套设施，改善城市交通。

　　增强城市集聚功能。坚持旧城改造与新城建设同步推进，深入实施“点线面”攻坚计划，进一步拉开框架、完善功能、美化环境、提升品位。南平中心城市围绕做大做强、做精做美的目标，开工建设延平新城的市政基础设施，加快建设高速铁路、高速公路连接线工程和水东新大桥，实施城区205、316国道改造，建成夏道大桥、滨江东路和闽江路，推进大家厂和湖尾城市综合体建设，引进一家大型商贸综合体，建设2家五星级饭店和2座公共停车场，改造提升一家四星级饭店，完成南平高速公路南北互通口景观改造提升和15公里绿道建设。各县域城市着重抓好“六个一”项目建设，整治一条主街道，开工建设一家以上大型城市商贸综合体，新建或改造提升一家以上高星级饭店，打造一条特色美食街，建成一条8公里以上绿道，建设一个产业专业园区，力争在年内取得明显成效。全面建成城市生活垃圾处理项目，加快城市污水配套管网建设，污水处理率达到80%以上。

　　加快建设美丽乡村。结合小城镇改革发展试点，推进绿色乡镇建设，各县（市、区）建成2个以上绿色试点村，推动宜居新村、绿色村庄和中心村镇融合发展。围绕新村居住社区化、就业渠道多元化、社会保障制度化、转移人口市民化、公共服务一体化的目标，加快旧村改造和新村建设步伐，大力推进农村环境卫生综合整治，完善农村基础设施，保护传统古民居和古树名木，建设农村风景林，优化农村人居环境，体现闽北农村山水风光和民居特色风格。

　　（五）全力建设武夷新区，培育区域发展增长极

　　打造绿色新区。围绕建设“世界遗产地、绿色生态城”，统筹考虑新区中心、武夷山、建阳、风景名胜、休闲度假旅游区和产业发展的功能布局，把绿色发展理念融入到规划设计、开发建设、招商选资的全过程。以武夷新区城市总体规划为指导，进一步优化规划设计，注重把握建筑风格外观，突出显山、露水、透绿，彰显新区绿色风貌和特色，努力把新区建设成为绿色发展的典范。

　　加快新区建设。继续打好“十大工程、百亿投资”战役，建成滨江西路等重要景观带、南林大道等骨干路网，加快建设省道303线武夷新区段改造工程和崇阳溪两岸景观带，迅速拉开城市框架。开工建设市民服务中心和一批统建房、酒店、公园、文化场馆等公建项目，逐步完善城市配套设施。推进厦门、泉州共建产业园建设，新引进重大产业项目30个以上、建成投产20个以上，新增规模工业产值10亿元以上。

　　创新体制机制。建立健全工程项目建设管理制度，确保规范、高效运作。加强与兴业银行、国开行、农发行等金融机构战略合作，积极争取债券融资，探索多种融资模式，有效解决新区建设资金问题。理顺武夷新区财税体制，形成市县一体推进新区开发建设的合力。

　　（六）大力保护青山绿水，持续加快建设生态市

　　构建绿色生态屏障。继续创建生态县（市、区）、生态乡（镇）、生态村，加快生态市建设步伐。做好高速公路、铁路、国省道两侧一重山和江河两岸的美化绿化，开展旅游景区的生态环境整治，加快推进福银高速公路南平段、延平至武夷山高速公路、205国道南平段绿色生态示范路建设，完成造林绿化47.5万亩。科学调整树种结构，加强生态公益林管护，严禁新上或扩建以天然阔叶树为原料的木材加工项目，继续严格控制木材采伐量，提高森林质量和生态功能。实施生态修复工程，深化矿山植被恢复和“青山挂白”治理，加强水土保持工作，完成40万亩水土流失综合治理。

　　加强自然资源保护。加强自然保护区、重要湿地、饮用水源等重点生态功能区的强制性保护，落实生态补偿政策。实行最严格的耕地保护制度，确保耕地占补平衡。开展土地矿产卫片执法检查，严格土地用途管制。开展低丘缓坡地开发利用试点工作，继续推进“三旧”改造，节约集约用地。

　　推进环境污染治理。继续深化闽江流域水环境综合整治，大力推进规模养殖场的全过程综合治理或生态种养改造。加强污染源头管理，实施脱硫脱硝并举、多污染物协同控制，加大烟粉尘治理力度，提高空气质量。加大节能减排力度，淘汰落后产能，发展循环经济，推行清洁生产。

　　（七）全面深化改革开放，激发创新发展新动力

　　推进重点领域改革。加强国有资产管理体制改革，做大做强国有集团公司。推进财税体制改革，开展营业税改征增值税试点。加快民营经济发展，拓宽民间投资的领域和范围，落实民营企业在融资、招投标、政府采购等方面的同等待遇。深化医药卫生体制改革，重点推进大病医疗保障机制和公立医院改革，探索组建医疗集团。

　　强化科技人才支撑。推动科技和经济紧密结合，着力构建以企业为主体、市场为导向、产学研相结合的技术创新体系。加快城市光网、4G网络和信息产业园区建设，促进“三网融合”，推进“数字南平”建设。完善科技创新服务平台，加强知识产权工作，举办科技成果交易会，支持龙头企业建设研发中心、设计中心、培训中心，提高企业自主研发能力，新增5家高新技术企业和创新型企业。创新人才工作机制，围绕新区开发、园区建设、产业发展和重点企业，加大招才引智力度，实施名校名医院带动战略，加强高层次人才培养、引进和使用，促进人才集聚。

　　扩大对外经贸合作。创新山海协作机制，积极参与闽东北经济协作区、闽浙赣皖九方经济区和泛珠三角经贸合作洽谈，落实南平与宁德区域合作框架协议，加快“飞地港”建设。加强与港澳台地区、国外友城的沟通联系，推进南台经贸文化交流合作，发挥南平旅港、澳门南平联谊会和闽北海外社团的作用，拓展对外开放领域。鼓励重点出口企业做大做强，增强出口产品竞争力。

　　（八）有效扩大公共服务，提高人民群众幸福感

　　办好惠民利民实事。建设20所公办幼儿园，消除2万平方米校舍的安全隐患，解决进城务工人员随迁子女就学问题，全面实施中等职业教育免费政策。新增25个群众性文化激情广场活动示范点，实现农村有线广播县乡村三级联播联控。加强市（县）医院建设，新增床位600张，城乡居民基本医保政府补助标准提高到280元。提高农村居民最低生活保障标准。实施保障性安居工程，建设各类保障性住房7200套、基本建成7000套。开展农村造福工程危房改造，搬迁2万人，建成10个以上集中安置区搬迁示范点。建设570个建制村志愿消防队，帮助部队建设生产生活项目。加强“餐桌污染”治理，建设“食品放心工程”。

　　提升社会保障水平。实施更加积极的就业政策，完善劳动就业社会保障公共服务平台，新增城镇就业2.3万人，转移农村劳动力就业5.2万人。推动城乡居民社会养老保险制度一体化，统筹做好城乡居民医疗、失业、工伤生育保险。加强老龄工作，筹建南平市老年活动中心。继续推进县级社会福利中心和农村敬老院建设。发展慈善、社会福利和残疾人事业，保障妇女和儿童合法权益，鼓励开展志愿服务活动。

　　加快发展社会事业。深入实施学前教育三年行动规划，强化中小学校标准化建设，大力发展优质高中和职业教育，全面实现“双高普九”，推进义务教育均衡发展。支持高等院校加快发展，提升职业院校办学水平，完成武夷学院本科教育合格评估。强化医疗卫生机构服务能力，促进基本公共卫生服务均等化，提高全民健康水平。广泛开展全民健身运动，不断提高群众身体素质。深化人口和计划生育综合改革，稳定低生育水平，提高出生人口素质。认真开展第三次全国经济普查。推进外事侨务、水文气象、方志档案等事业发展。

　　促进文化繁荣发展。加强社会主义核心价值体系建设，大力弘扬福建精神，深入学习宣传詹红荔、朱邦月等先进典型，提高群众性精神文明创建实效，不断聚合社会主义文化繁荣发展正能量。加强武夷岩茶制作等非物质文化遗产保护与传承，扶持文艺精品创作，打造朱子文化、武夷茶道、闽越文化、大圣文化等闽北特色文化品牌。推进有线数字电视整体转换和广电光缆联网，完善公共文化服务体系。抓好重点文化产业项目建设，发展壮大文化产业。

　　加强社会管理创新。健全社会稳定风险评估和应急处置机制，完善信访制度和“大调解”工作体系，推广镇村组矛盾化解“三级联动”和“六要”群众工作法，完善矛盾纠纷专业调解组织，切实把不稳定因素化解在源头。推进社会管理网格化，全面开展平安先行县（市、区）、平安先行单位及行业创建活动，开展“六五”普法，深化综合警务改革创新，完善立体化社会治安防控体系，深化“打四黑、除四害”和“扫黄打非”等专项行动。全面推进企业安全生产标准化达标创建工作，继续实施交通安全综合整治三年行动方案，加强生产安全、校园安全、消防安全、食品安全、社会安全的监督管理。落实民族政策，依法管理宗教事务。支持工会、共青团、妇联等人民团体和社会组织开展工作。深化“双拥”共建，强化国防教育、国防动员、国防后备力量和人民防空建设。

　　三、加强政府自身建设

　　加快绿色发展推进生态文明建设，对政府工作提出更高的要求。各级政府要以科学发展观为指导，大力加强自身建设。

　　（一）解放思想，提升作为。坚持以党的十八大精神武装头脑，加强对各项政策和现代知识的学习研究，进一步解放思想、更新观念，不断提高领导科学发展、驾驭复杂局面的能力。坚持以推动南平绿色发展为己任，发扬滴水穿石、人一我十的精神，自加压力，攻坚克难，奋力拼搏，力求在工作中实现更大的进位。

　　（二）转变职能，优化服务。深化政府机构改革，积极稳妥推进事业单位分类改革，做到不缺位不越位不错位。深入推进行政审批改革，推行项目联审和集中审批，缩短流程链条，简化办事程序，倡导“马上就办”，提高办事效率。强化效能督查，完善绩效评估，加大治庸治懒治散治奢的力度，建设服务型政府。

　　（三）改进作风，务实为民。严格按照中央“八项规定”要求，倡导“实”的作风、创新“抓”的方法、树立“干”的导向，推行一线工作法，强化目标约束，严格督查问责，落实领导责任，提高政府执行力。深入开展“我和百姓拉家常”和党的群众路线教育实践活动，多做惠民生、谋民利和打基础、利长远的实事好事，维护人民群众的合法权利。

　　（四）依法行政，从严治政。主动接受人大依法监督、政协民主监督，自觉接受社会监督和舆论监督，强化行政监察和审计监督。落实党风廉政建设责任制，全面推进惩治和预防腐败体系建设。坚持勤俭办事业，严格控制“三公经费”等一般性支出。加强政务公开，依法依规做好土地出让、资产处置、政府采购等公共资源交易事项，推进权力运行公开化、规范化。

　　各位代表，我们要紧密团结在以习近平同志为总书记的党中央周围，在省委、省政府和市委的正确领导下，深入学习贯彻党的十八大精神，凝聚全市人民的智慧和力量，坚定信心，开拓进取，奋勇争先，加快绿色发展推进生态文明建设，为建设充满活力、富裕文明、和谐安康、幸福美好的南平而努力奋斗！
